# Gene Expression Regulation in Airway Pathogens: Importance for Otitis Media

**DOI:** 10.3389/fcimb.2022.826018

**Published:** 2022-02-11

**Authors:** Martina Janoušková, Megan Laura Straw, Yu-Ching Su, Kristian Riesbeck

**Affiliations:** Clinical Microbiology, Department of Translational Medicine, Faculty of Medicine, Lund University, Malmö, Sweden

**Keywords:** gene expression regulation, *Haemophilus influenzae*, *Moraxella catarrhalis*, otitis media, *Streptococcus pneumoniae*

## Abstract

Otitis media (OM) is an inflammatory disorder in the middle ear. It is mainly caused by viruses or bacteria associated with the airways. *Streptococcus pneumoniae, Haemophilus influenzae* and *Moraxella catarrhalis* are the three main pathogens in infection-related OM, especially in younger children. In this review, we will focus upon the multifaceted gene regulation mechanisms that are well-orchestrated in *S. pneumoniae, H. influenzae*, and *M. catarrhalis* during the course of infection in the middle ear either in experimental OM or in clinical settings. The sophisticated findings from the past 10 years on how the othopathogens govern their virulence phenotypes for survival and host adaptation *via* phase variation- and quorum sensing-dependent gene regulation, will be systematically discussed. Comprehensive understanding of gene expression regulation mechanisms employed by pathogens during the onset of OM may provide new insights for the design of a new generation of antimicrobial agents in the fight against bacterial pathogens while combating the serious emergence of antimicrobial resistance.

## Introduction

Otitis media (OM) covers a spectrum of middle ear (ME) inflammatory disorders that are caused by various irritating agents and pathogens. In low to middle-income countries, OM is the main medical condition for antibiotics prescription and surgeries, and deafness in children, respectively. Despite OM is a relatively mild condition that in many cases heals spontaneously, it can cause severe illness and thousands of OM-related deaths have been reported annually (Monasta et al., 2012; Schilder et al., 2016).

**Figure 1:**
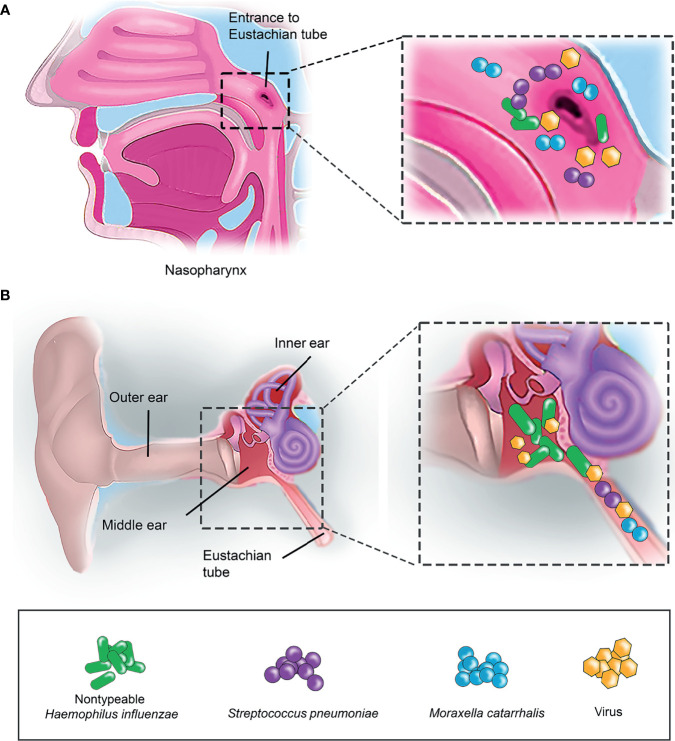
Bacterial colonisation in human nasopharynx and middle ear. **(A)** Nasopharynx is the primary entrance for airway pathogens. Small insert (left panel) indicates the enlarged view of the entrance to Eustachian tube in nasopharynx. Airway pathogens such as nontypeable *H*. *influenzae* (NTHi) (indicated as green rod)*, S. pneumoniae* (Spn) (purple sphere) and *M. catarrhalis* (Mcat) (blue sphere) that initially colonize the nasopharynx as part of the commensal microbiota can, however, migrate into the middle ear (ME) *via* the Eustachian tube. **(B)** Co-infection of airway pathogens and viruses in the ME. Airway pathogens (NTHi, Spn and Mcat) that have successfully entered the Eustachian tube can travel into the ME and colonize as a biofilm. This results in a middle ear inflammation triggered by the host immune response, and subsequently leads to the onset of OM. Virus (yellow hexagon) infection is often preceding the bacterial infection in OM.

*Streptococcus pneumoniae* (Spn), non-typeable *Haemophilus influenzae* (NTHi), and *Moraxella catarrhalis* (Mcat) are the three main othopathogens isolated from OM patients (Biesbroek et al., 2014; Mills et al., 2015; Lee et al., 2020). The course of othopathogen-dependent OM involves bacterial colonization on the ME epithelium (Hendrixson and St Geme, 1998; Thornton et al., 2011). Bacterial infection in the ME usually occurs as a secondary infection to viral infection in the nasopharynx that subsequently progresses to ME *via* the Eustachian tube (Jossart et al., 1994; Jiang et al., 1999) (**Figure 1**). According to general guidelines in various countries, antimicrobial agents, depending on the age group of children are the treatment of choice of OM. An effective vaccine for bacterial-related OM is still unavailable (Jalalvand and Riesbeck, 2018). Child immunization programs including conjugated pneumococcal vaccines have, however, decreased the incidence of Spn-related OM in most countries (Littorin et al., 2016; Littorin et al., 2021).

To overcome the hostile environment in the human airway during OM, bacteria are forced to have sophisticated gene regulation expression machineries for optimum survival and adaptation especially in the nasopharynx and ME. In this review, we will mainly focus on the most studied gene regulation mechanisms of Spn, NTHi and Mcat that are associated with OM in the past decade.

### Streptococcus pneumoniae

*Streptococcus pneumoniae* is a Gram-positive diplococcus with capsulated cell wall (Brooks and Mias, 2018). The bacteria asymptomatically colonize (carriage phase) the human upper airway as a part of commensal microbiota in a healthy individual. The carriage phase is essential for pneumococcal pathogenic transition to other sterile sites causing symptomatic infections, such as OM in the ME, in individuals with an immature or weakened immune system (Chao et al., 2014; Coleman et al., 2018; Weiser et al., 2018).

The transition of Spn from nasopharyngeal colonization to ME infection is a multifactorial process. The pathogen needs to adapt to the diverse environmental conditions in the human airway during transition between niches. This includes the varying levels of nutritional status, acidities, oxygen, carbon dioxide, temperature, and a myriad of host antimicrobial factors (Kloosterman and Kuipers, 2011; Manzoor et al., 2015; Paixao et al., 2015a; Paixao et al., 2015b; Man et al., 2017; Aprianto et al., 2018). The establishment of Spn colonization in the nasopharynx and ME requires fine-tuned gene expression of a plethora of virulence and metabolism factors in response to the environmental conditions at target niches. Pneumococcal virulence factors, as recently reviewed in detail by Brooks *et al.* and Weiser *et al*., promote biofilm formation and contribute to pneumococcal adherence to airway epithelial cells, evasion of mucosal clearance by the host immune system, and to outcompete co-colonizing bacteria or resident flora (Brooks and Mias, 2018; Weiser et al., 2018).

### Gene Expression During Colonization

Besides the host immune factors, virus infection, the co-colonizing microbiome, and exposure to inhalable particle matters at the nasopharynx and ME can also promote Spn infection in the ME. Host norepinephrine, extracytoplasmic ATP, and nutrients (*i.e.*, N-acetylneuraminic acid and N-acetylglucosamine) released from the damaged nasopharyngeal tissue following a simultaneous viral infection promote pneumococcal dispersal from nasopharyngeal biofilm colonization and migration to the ME (Marks et al., 2013; Chao et al., 2014; Paixao et al., 2015a; Paixao et al., 2015b; Aprianto et al., 2018). Biofilm-dispersing Spn exhibit aggressive growth and virulence phenotypes through their increased production of bacteriocins, virulence factors (capsule, *cbp*A, *psp*A, *ply*, *pcp*A, *nan*A, and *nan*B), proteins of unknown function (SPV_2027 and SPV_2171), and factors related to carbohydrate metabolism, while reducing the expression of genes for competence and adhesion (Gualdi et al., 2012; Allan et al., 2014; Pettigrew et al., 2014; Paixao et al., 2015a; Paixao et al., 2015b; Aprianto et al., 2018). Particle matters (*i.e.*, Asian sand dust, cigarette smoke and black carbon source) induce biofilm formation hence Spn colonization of the human ME epithelium in cell lines and in the ME of mouse OM model (Hussey et al., 2017; Yadav et al., 2020). Genes for biofilm formation (*luxS*), competence (*comA, comB, ciaR*), toxin production (*lytA* and *ply*), detoxification, efflux pumps and osmo-regulator transporters were up-regulated during pneumococcal infection (Cockeran et al., 2014; Manna et al., 2018; Yadav et al., 2020).

### Mechanism in Gene Regulation: Pheromone Peptide Signaling

**Figure 2:**
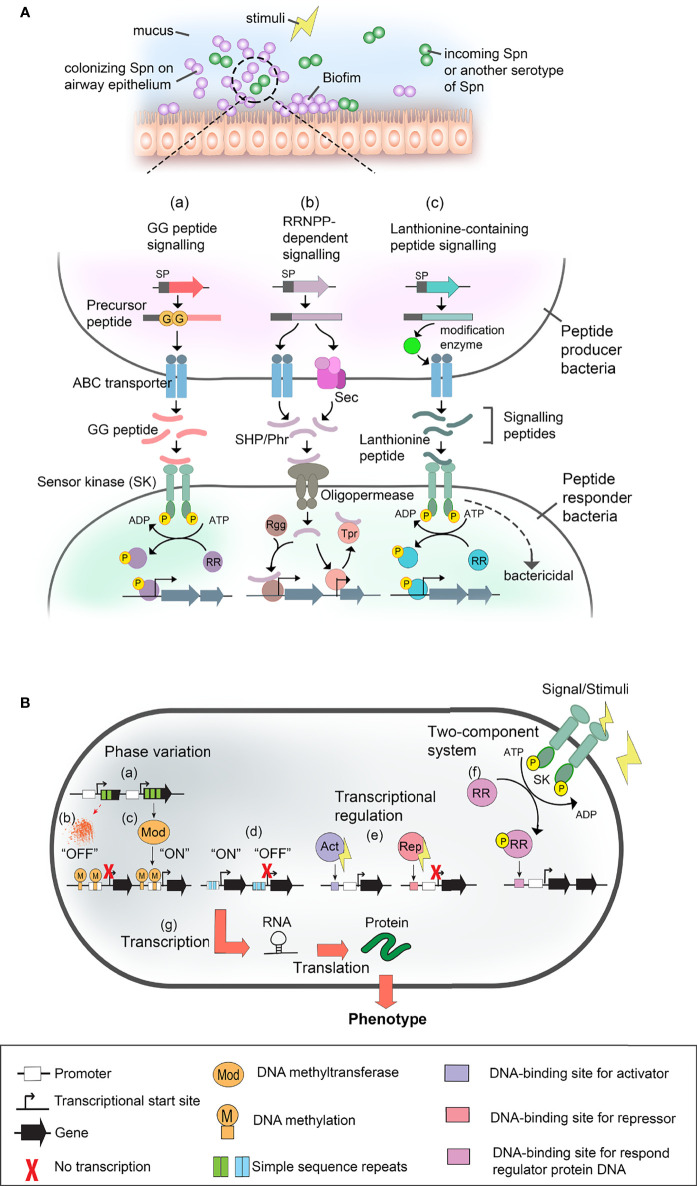
Gene expression regulatory system in *S. pneumoniae* (Spn), and nontypeable *H*. *influenzae* (NTHi), and *M. catarrhalis* (Mcat). **(A)** Pneumococcal cell-cell communication systems. Spn colonizing the upper airway mucosa (*i.e.*, at nasopharynx and middle ear) form biofilm and interact with either neighbouring pneumococcal or incoming clone of Spn *via* quorum sensing or cell-cell communication in response to surrounding stimuli (upper panel). Pheromone peptide signalling pathways and their regulatory effect in gene expression during Spn cell-cell communication are shown in the lower panel. Pheromone peptides such as double glycine peptides [GG peptide (*i.e.*, CSP, BriC, VP1)] [as shown in pathway (a)], RRNPP-dependent peptides (*i.e.*, SHR and Phr) [shown in (b)], and lanthionine peptides (*i.e.*, pneumolancidin PldA1-A4) (shown in (c)) are secreted by pneumococci and autoinduced in response to stimuli. Depending on the signal sequence (SP) of the peptides, they are exported *via* ABC transporter or general secretory (SEC) pathway. The precursor peptide is proteolytically processed into active peptide either during transportation by peptidase domain of ABC transporter or membrane-associated proteases, or after secretion *via* unknown extracellular protease. At a sufficient level of the signalling peptide pheromone, they interact with their cognate cell receptors in recipient cells (peptide responder bacteria) such as sensor kinase (SK) of two-component system (TCS) [shown in pathway (a) and (c)]. In canonical TCS, the sensor protein is a histidine kinase (*i.e.*, ComAB, BlpAB and PptAB) that detects exogenous signals, and subsequently, sends a phosphoryl group (yellow sphere) to the cognate response regulator protein (RR). The phosphorylation of the regulator protein results in transcriptional regulation. Alternatively, some peptides such as bacteriocin CibAB and lanthibiotics (pneumolandin) can activate recipient cell directly and induce bactericidal effect. As shown in pathway (b), for RRNPP-dependent peptides such as SHP (*i.e.*, SHP144, SHP939 and RgtS) and Phr (*i.e.*, PhrA), they are transported into responder cell *via* oligopeptide permease system (*i.e.*, AmiACDEF). Once inside the recipient cell, SHPs interacts with their cognate binding partner, Rgg regulators (*i.e.*, Rgg144, Rgg939, abd RgtR), resulting in DNA binding of Rgg and activation of transcription. On the other hand, Phr peptides interact with Tpr regulators that are initially bound to DNA and inhibit the expression of the target gene. Binding of Phr to Tpr results in the releasing of Tpr-mediated inhibition hence activates gene expression. Lastly, lanthionine-containing peptide such as pneumolandin (*i.e.*, PldA1-A4) is processed and translationally modified by intracellular modification enzyme such as LanM before transportation *via* ABC transporter (*i.e.*, LanT) and detected by SK of responder cells (*i.e.*, LanA) [shown in pathway (c)]. This results in either phosphorylation of the response regulator for downstream activation of gene expression, or directly causing bactericidal effect. ABC transporter, ATP-binding cassette transporter; Phr, phosphatase regulator; Rgg, regulator gene of glycosyltransferase; RRNPP, Rap, Rgg, NprC, PlcR and PrgX; SHP, short hydrophobic peptide. **(B)** Schematic representation of gene regulatory mechanism in NTHi and Mcat. Phase variation [shown in (a)] is caused by random mutations in a variable number of simple sequence repeats (SSR) within the open reading frame of DNA-methyl transferases (*i.e.*, ModA and ModM] that alters the gene expression of Mod. Thus, any changes in the variable number of the SSRs as a result of DNA mutation could cause frame shifts in the ORF of *mod*A and potentially lead to premature translation termination and generation of non-functional truncated ModA which is in the “OFF” mode (shown in (b)]. On the other hand, the functional Mod (switched “ON”) [shown in (c)] methylates genomic DNA at specific sites that governs transcription in both ways, either by inhibition or activation. The inhibition or activation of transcription depends on the methylated DNA sequence area that potentially bears the recognition sites for regulatory molecules and enzymes. Moreover, the phase-variable number of SSRs [as shown in (d)] can also be found in the transcriptional promoter which, as a result, switches between the “ON” or “OFF” expression status of the target gene (*i.e., hia*). In addition, transcriptional regulators [shown in (e)] can be activators (Act) (*i.e.*, OxyR, Fur) and/or repressors (Rep) (*i.e.*, Fur) of transcription. The regulators are triggered by various stimuli. (f) Another type of transcriptional regulation is a TCS (*i.e.*, FirRS, mesSR, narX/narL) which consists of sensor kinase (SK) and response regulator proteins (RR). The activated regulatory proteins can cause transcription activation and/or repression as described in (e). Finally [indicated in (g)], the genes are transcribed into RNA, followed by translation as protein. Alterations in gene expression are manifested in a bacterial phenotype. Symbols used in panel **(A, B)** are defined at the bottom of this figure.

Gene expression in Spn, in response to environmental stimuli, is regulated and synchronized at a population-level through quorum sensing or cell-cell communication systems that are orchestrated by short peptide pheromone-mediated signaling pathways. Three main types of pneumococcal cell-cell communication systems have been discovered so far. They are either mediated by (i) glycine-glycine (GG) peptides, (ii) peptides that signal *via* RRNPP superfamily regulators, or (iii) lanthionine-containing peptides, as depicted in **Figure 2A**. The most well-studied pneumococcal GG peptide signaling is the competence-stimulating peptide (CSP) that is autoinduced hence actively secreted when the surrounding pH, hemoglobin, oxygen levels, antimicrobial stress, and, finally, the bacterial cell density is high (Gagne et al., 2013; Slager et al., 2014; Domenech et al., 2018; Weyder et al., 2018; Paton and Trappetti, 2019; Akhter et al., 2021). A sufficient level of the GG peptide eventually signals the neighboring recipient pneumococci *via* a two-component system (TCS) and alter their gene expression. The CSP signaling is responsible for the induction of (i) competence and transformation in Spn contributing to genetic diversity or as nutrient source; (ii) bacteriocin peptides (CibAB, peptides of the bacteriocin immunity region (BIR), and bacteriocin-inducing peptide (BIP)) for microbial competition; and (iii) biofilm-regulating peptide induced by competence (BriC) that induces the biofilm formation and nasopharyngeal colonization in animal models while altering the fatty acid biosynthesis in the pneumococcal membrane homeostasis for increased adaptation in the host (Valente et al., 2016; Wholey et al., 2016; Aggarwal et al., 2018; Wang et al., 2018; Aggarwal et al., 2021).

Due to a low availability of glucose in the airways, Spn in the nasopharynx and ME relies on the galactose and mannose derived from the airway mucosal glycan lining, as a main carbon source for energy metabolism and virulence (Paixao et al., 2015a; Paixao et al., 2015b). Several recent studies have unveiled the impact of carbohydrate utilization and metabolic processes in Spn virulence, persistence, and infection at nasopharynx, which involves a series of regulators (*gal*K, *gal*R, *hyl, ugl, lacD, nanA, eng*, *raf*K, *est*A, and auto-inducer AI-2) (Afzal et al., 2015; Mclean et al., 2020; Minhas et al., 2021). Sensing of the host carbohydrates for environmental adaptation is mainly carried out by pneumococcal short hydrophobic peptide (SHP) and phosphatase regulator (Phr) that interact with RRNPP superfamily of transcription regulator, regulator gene of glycosyltrasferase (Rgg) and transcription factor regulated by Phr peptide (Tpr), respectively. High abundance of galactose and mannose stimulates SHP144 and SHP939 to autoinduce, whereas induction of PhrA is more related to galactose dependence (Junges et al., 2017; Zhi et al., 2018; Motib et al., 2019). SHP144 and SHP939 imported into the recipient neighboring pneumococci positively regulate their cognate regulator, Rgg144 and Rgg939, respectively. This results in the upregulation of regulons involved in environmental adaptation (*i.e.*, genes for replication and translation, nucleotide metabolism, cell division, ion transport, and capsule production) (Junges et al., 2017; Zhi et al., 2018; Cuevas et al., 2019). The SHP144/Rgg144 signaling also positively regulates the transcription of VP1, a novel virulent GG-peptide of Spn that was highly expressed in chinchilla ME effusions (Cuevas et al., 2017; Cuevas et al., 2019). VP1 activates biofilm development, colonization, hyaluronic acid-dependent attachment of Spn. On the other hand, PhrA imported into the recipient pneumococci inhibits the repressor role of its cognate regulator TprA, activating the transcription of genes for sugar metabolism, neuraminidase activity, and locus of lanthionine-containing peptide for microbial competition during colonization (Hoover et al., 2015; Motib et al., 2017; Motib et al., 2019). Lantibiotic-containing peptides (pneumolancidins, Pld) are another class of pneumococcal bacteriocins that can be autoinduced by histidine kinase receptor signaling in response to the carbon source and cell density (Hoover et al., 2015; Maricic et al., 2016). This ultimately benefit pneumococci to outcompete co-colonizing bacteria for space and nutrients during colonization.

LuxS/AI-2-dependent quorum sensing system is also another crucial gene regulatory system in Spn especially for biofilm formation, genetic competence, and fratricide (Trappetti et al., 2011; Yadav et al., 2018). The transcriptomic and mutagenesis studies revealed that LuxS is the central regulator of genes important for Spn virulence and persistence in carriage phase and middle ear infections.

### Non-Typeable *Haemophilus influenzae*

Non-typeable *Haemophilus influenzae* (NTHi) is a Gram-negative coccobacillus without capsule that is genetically non-clonal (Erwin et al., 2005; Whittaker et al., 2017). NTHi is part of the nasopharyngeal commensal microbiota. The bacterial species can also, however, cause airway infections such as OM. The currently used vaccine specific to *H. influenzae* type b (Hib) is not effective against NTHi. The pathogen is heme and nicotinamide adenine dinucleotide (NAD) auxotroph, hence requires exogenous supplementation of these elements for growth (Jalalvand and Riesbeck, 2018; Su et al., 2018).

### Gene Expression During Colonization

Similarly to Spn, NTHi colonizing the human airway needs to overcome the harsh environmental conditions in the host in order to establish a fulminant infection. Proteomic analysis on NTHi infecting the chinchilla ME revealed the altered expression of 28 bacterial proteins involved in carbohydrate and amino acid metabolism, redox homeostasis, and cell wall-associated metabolic proteins (Harrison et al., 2016). This implies the utilization of glucose by NTHi for aerobic respiration during animal AOM.

Inflammation triggered in the ME upon infection leads to activation of nutritional immunity (Szelestey et al., 2013). This results in the sequestration and restriction of free metal ions available for NTHi iron/heme-dependent metabolic enzymes, regulatory proteins, and aerobic respiration that are essential for bacterial ME colonization (Szelestey et al., 2013; Harrison et al., 2016). Since NTHi is heme-iron auxotroph, the pathogen develops a plethora of iron/heme acquisition mechanisms in response to the host nutritional immunity. This includes the upregulation and expression of a series of core iron- and heme-responsive genes which some are regulated by the NTHi ferric uptake regulator (Fur) (**Figure 2B**) and RNA chaperon Hfq (Harrison et al., 2013; Hempel et al., 2013; Whitby et al., 2013). This in turn promotes the colonization persistence and virulence factor expression in NTHi when infecting the chinchilla ME. Besides the iron uptake genes, Fur also regulates the transcription of small RNA HrrF that is important for molybdate uptake, deoxyribonucleotide synthesis and amino acid biosynthesis (Santana et al., 2014). The high abundance of iron/heme also activates the promoter activity of *pil*A, hence the expression of subunit PilA for the type IV pilus that is essential for NTHi adherence and biofilm formation (Mokrzan et al., 2019). Moreover, the co-culture with Spn enhances *pilA* expression in NTHi and biofilm formation as well (Cope et al., 2011). Biofilm formation is crucial for NTHi colonization in OM, and the biofilm is also regulated by a TCS transduction system named QseBC/FirRS (ferrous iron responsive regulator/sensor) (**Figure 2B**), which is responsive to low temperature and availability of nutrients (ferrous iron and zinc) (Steele et al., 2012; Unal et al., 2012; Van Hoecke et al., 2016). In addition, high pH in the ME during OM promotes biofilm formation and expression of virulence factors for iron acquisition (Ishak et al., 2014).

Beside the nutritional immunity, NTHi also needs to combat oxidative stress such as reactive oxygen species (ROS) and reactive nitrogen species (RNS) generated by the airway immune defense. Hence, NTHi upregulated the expression of OxyR regulon (antioxidant enzymes (peroxiredoxin (PgdX) and catalase (HktE)) as shown in the chinchilla ME (Whitby et al., 2012; Parrish et al., 2019).

### Mechanism in Gene Regulation: Phase Variation

Phase variation is one of the most studied gene regulation mechanisms in NTHi. It is widely used by NTHi to regulate the expression of virulence factors that are crucial for bacterial colonization at specific niches. Phase variation is a random molecular event that enable a specific gene expression to be reversibly switched “ON” and “OFF”. This mechanism is based upon the DNA mutation that results in the formation of variable number of simple sequence repeats (SSR) in the genome (Srikhanta et al., 2005) (**Figure 2B**).

In NTHi, N6-adenine DNA-methyltransferase (ModA) is one of the proteins that have a phase variable expression. The enzyme which is part of the type III restriction-modification (R-M) system methylates bacterial chromosomal DNA at a specific site on the genome, mediating epigenetic regulation (Gawthorne et al., 2012). ModA is encoded by a *mod*A allele. Up to 21 allelic variants of *mod*A (*modA*1-21) exist in NTHi clinical isolates taken from COPD patients and children with ME infection or OM (Atack et al., 2015a; Atack et al., 2019). However, among the identified *mod*A allelic variants, 65% of the isolates carry one of just 5 phase-variable *mod*As, namely allelic variant of *modA*2, 4, 5, 9, or 10 (Fox et al., 2007; Atack et al., 2015a). These alleles contain different numbers of SSRs and hence can alter the expression of ModA. The phase variation of these alleles impacts (i) the gene expression of (a) known NTHi virulence major outer membrane proteins (P2, P5, P6, and HMW) (*modA*2, 4, 5, 9, and 10-dependence) and (b) NTHi proteins involved in antibiotic resistance (mod*A*2, 5 and 10), and (ii) evasion of opsonophagocytic killing (*mod*A4).

In response to the alkaline pH environment occurred in the ME during OM, ModA2 of NTHi mediates the formation of a bacterial biofilm that has greater biomass and stable structure which are critical for NTHi pathogenesis *in vivo* (Atack et al., 2015a; Brockman et al., 2018). NTHi with phase variable *mod*A2 expression (reversibly switch between “ON” and “OFF”) has survival and adaptational advantages for colonization at chinchilla ME over an isogenic strain that is unable to phase vary their ModA2 expression. This is explained by the fact that NTHi carrying *mod*A2 that is permanently “OFF” or “ON” is more susceptible to macrophage and neutrophil killing, respectively; whereas strains carrying phase variable *mod*A2 (switchable from “OFF” to “ON”) may alter their virulence factor expression for increased antigenic variation hence evasion of the host defense (Brockman et al., 2016; Robledo-Avila et al., 2020). Collectively, the methylation-dependent gene regulation mediated by *mod*A influences the virulence of NTHi especially during infection at ME. In addition, to avoid the host immune system during invasive infection, NTHi also alters the expression of Hia, an immunogenic adhesin, by changing the length of polythymidine tract on the *hia* promoter (Atack et al., 2015b). SSR-dependent phase variation is also used by NTHi for persistence and adaptation in the pathogenesis of other airway infections such as COPD (Poole et al., 2013; Pettigrew et al., 2018; Fernandez-Caldet et al., 2021).

### Moraxella catarrhalis

*Moraxella catarrhalis* is a Gram-negative respiratory opportunistic pathogen. It is categorized into two distinct lineages based on 16S rRNA sequence: (i) ribotype (RB) 1 which comprise of ∼80-90% of isolates associated with adherence and serum resistance; (ii) RB2 and RB3 strains that are detected in ∼10-20% isolates (Bootsma et al., 2000; Verhaegh et al., 2011).

### Gene Expression During Colonization

Regulation in gene expression of virulence factors is crucial for Mcat colonization and long-term survival within the host in the human airways. There are a range of extrinsic stimuli that this pathogen must contend with, such as low temperature and iron restricted conditions within the human airway. The environmental factors affect the expression of Mcat major outer membrane proteins that are the key to efficient colonization (Jetter et al., 2010; Spaniol et al., 2011). Notably, genes of resistance-nodulation-division (RND) multidrug efflux systems (*acr*AB and *opr*M) are upregulated at nasopharyngeal temperature (26°C) (Spaniol et al., 2013). Deletion of *acrAB* and *opr*M caused Mcat to lose up to 50% invasion capacity on human pharyngeal epithelial cells (Spaniol et al., 2015).

PilA that mediates Mcat *in vivo* colonization in the chinchilla model was the earliest adhesin to be investigated (Luke et al., 2007). Hoopman *et al*., further elucidated the Mcat colonization *via* DNA microarray analysis which showed the upregulated genes involved in the oxidative stress response, denitrification pathway, in addition to an uncharacterized gene (ORF1550) that is crucial for Mcat persistence (Hoopman et al., 2012). Other well-known adhesin-encoding virulence genes, such as *uspA1*, *uspA2*, *uspA2H*, *mid/hag* and *mcaP*, *mha/mch* and *mcm* also play important roles in protecting Mcat from the serum bactericidal effect by interfering with the host complement pathway factors (Singh et al., 2016; Riesbeck, 2020). A mutation study revealed the impact of an outer membrane lipoprotein (ORF113) on the survival of Mcat in chinchilla (Wang et al., 2014).

Several Mcat virulence factors are growth phase dependent. The strain Mcat RH4 displayed increased expression of *mid/hag* during the lag and stationary phases but fell to lower levels during exponential phase (Riesbeck and Nordstrom, 2006; De Vries et al., 2010). MID/Hag protein is probably essential for initial colonization but not for proliferation. In a separate study, the downregulated expression of *mid/hag* and *uspA2* occurred during infection in chinchilla (Hoopman et al., 2012). MID/Hag expression was also reduced during persistent colonization of Mcat in COPD patients (Murphy et al., 2019). It appears that reducing the profusion of surface proteins could aid Mcat to avoid host recognition.

One of the main modes of Mcat colonizing the ME during infection is *via* biofilms. The synergistic relationship Mcat has with its co-colonizing pathogens increases OM incidence (Blakeway et al., 2018). Mcat secretes beta-lactamase-containing outer membrane vesicles (OMVs) in biofilms which protects co-colonizing pathogens from beta-lactam antibiotics (Armbruster et al., 2010; Schaar et al., 2013). There are interesting facets of biofilm formation which include the quorum-sensing systems found in Spn, NTHi and Mcat. Armbruster *et al*. established the compensation effect of the NTHi *lux*S gene (interspecies quorum signaling factor) in aiding Mcat for persistence in chinchilla since the latter species does not possess a *lux*S homolog (Armbruster et al., 2010; Mokrzan et al., 2018).

### Mechanism in Gene Regulation: Phase Variation and Two-Component System

Once Mcat has colonized the host, bacteria regulate the expression of its cell surface components by phase variation. The phase variation mechanism that has been widely studied in Mcat is performed by several DNA methyltransferases including ModM. The DNA methyltransferases are part of R-M system that master mind the gene regulation in Mcat during infection (Blakeway et al., 2018; Phillips et al., 2019) (**Figure 2B**).

Similar to NTHi, Mcat also carries multiple allelic variants of *mod*M (*mod*M1-6). The most common allele of *mod*M present in Mcat isolates is *mod*M2 which regulates genes associated with colonization and host immune evasion (Blakeway et al., 2019). Although the *mod*M3 allele is commonly found within the minor lineage RB2 and RB3 of Mcat strains, the ModM3 methyltransferase is prevalent in Mcat clinical isolates taken from children with OM (Blakeway et al., 2014). Extensive methodologies were used including single molecular real-time (SMRT) methylome analysis to ascertain the methylation activity of ModM3. Also, RNASeq analysis revealed that ModM3 alters the expression of genes involved in biofilm formation, anaerobic tolerance, nitrosative and oxidative stress responses. Specifically, in response to host immune defense-derived nitrosative and oxidative stress, Mcat with “ON” variant of *mod*M3 upregulated the expression of genes *narX/narL*, and a predicted AhpC/TSA family peroxiredoxin RS03200, respectively (Blakeway et al., 2019). Additionally, in a recent study aiming to reveal the correlation between serum resistance and phase variation, Mcat strains that survived in human serum have higher mRNA levels of *uspA2* whereas *mid/hag* and *uspA1* showed reduced expression over a longer time of exposure to the human serum (Tan et al., 2020). Under this selection pressure, variable SSR tract lengths that regulate transcription hence phase variation in *uspA1, uspA2* and *mid/hag* is likely to occur. Antigenic variation of outer membrane proteins is another strategy of Mcat for host immune evasion (Murphy et al., 2019; Tan et al., 2020).

Another gene regulatory mechanism of Mcat is the TCS signaling systems (**Figure 2B**). *mesR* is a gene of this signalling system, and potentially regulates expression of two lysozyme inhibitor genes as a defence against the host immune response (Joslin et al., 2015). Another example of TCS is the *narX/narL* involved in denitrification pathway. This system counteracts reactive nitrogen species produced exogenously and could possibly ensure Mcat survival under anaerobic conditions (Blakeway et al., 2019).

## Conclusion

The ability to overcome environmental challenges during colonization inside the host is crucial for bacterial survival and persistence, hence establishment of infection. Spn senses the environmental stimuli and regulates its gene expression through short peptide pheromone-mediated signalling pathways and LuxS/AI-2-dependent quorum sensing system. This enables Spn to regulate the switch between biofilm and planktonic phenotype for colonization or invasion, respectively; genetic competence, and fratricide activities, and expression of regulons involved in environmental adaptation and evasion of host immune system. On the other hand, in addition to transcriptional regulators, gene regulation in NTHi and Mcat is mainly mediated *via* phase variation mechanisms that reversibly switches the expression “ON” or “OFF” of the target genes. The system is based on the presence of the variable number of SSRs, as a result of slipped-strand mispairing that occurred either on the promoter or within the ORF, or both, of the target gene. This enables NTHi and Mcat to regulate their biofilm formation, adherence, serum resistance and other virulence phenotype according to growth phase or in response to selective pressure caused by host immune response. The findings discussed in this review could help to improve our understanding regarding the gene expression governing system in these pathogens and can be a potential target of future antimicrobial intervention strategy against Spn, NTHi and Mcat infection.

## Author Contributions

MJ coordinated the manuscript. MJ, MS, and Y-CS drafted the manuscript. Literature studies of virulence and gene regulation in NTHi, *M. catarrhalis* and *S. pneumoniae* were carried out by MJ, MS, and Y-CS, respectively. Figures were mainly prepared by MJ and MS assisted by Y-CS. All authors edited, critically revised, and approved the final manuscript.

## Funding

We thank the following funding agencies for their financial support during the preparation of the manuscript. They are, the Swedish Medical Research Council (KR: grant number 2019-01053, www.vr.se), the Anna and Edwin Berger Foundation (KR), the Physiographical Society (Forssman’s Foundation; MJ, MS), Skåne County Council’s research and development foundation (KR), and Heart Lung Foundation (KR: grant number 20180401, www.hjart-lungfonden.se).

## Conflict of Interest

The authors do not have any commercial interests. KR is participating in projects supported by Pfizer and has been collaborating with GSK.

The remaining authors declare that the research was conducted in the absence of any commercial or financial relationships that could be construed as a potential conflict of interest.

## Publisher’s Note

All claims expressed in this article are solely those of the authors and do not necessarily represent those of their affiliated organizations, or those of the publisher, the editors and the reviewers. Any product that may be evaluated in this article, or claim that may be made by its manufacturer, is not guaranteed or endorsed by the publisher.
